# The DNA Repair Protein OGG1 Protects Against Obesity by Altering Mitochondrial Energetics in White Adipose Tissue

**DOI:** 10.1038/s41598-018-33151-1

**Published:** 2018-10-05

**Authors:** Sai Santosh Babu Komakula, Jana Tumova, Deeptha Kumaraswamy, Natalie Burchat, Vladimir Vartanian, Hong Ye, Agnieszka Dobrzyn, R. Stephen Lloyd, Harini Sampath

**Affiliations:** 1 Department of Nutritional Sciences and Rutgers Center for Lipid Research, New Jersey Institute for Food, Nutrition, and Health, Rutgers University, New Brunswick, NJ 08901 USA; 2 Laboratory of Cell Signaling and Metabolic Disorders, Nencki Institute of Experimental Biology, Warsaw, Poland; 3 Oregon Institute of Occupational Health Sciences, Department of Molecular and Medical Genetics, Oregon Health & Science University, Portland, OR 97239 USA

## Abstract

Obesity and related metabolic pathologies represent a significant public health concern. Obesity is associated with increased oxidative stress that damages genomic and mitochondrial DNA. Oxidatively-induced lesions in both DNA pools are repaired via the base-excision repair pathway, initiated by DNA glycosylases such as 8-oxoguanine DNA glycosylase (OGG1). Global deletion of OGG1 and common OGG1 polymorphisms render mice and humans susceptible to metabolic disease. However, the relative contribution of mitochondrial OGG1 to this metabolic phenotype is unknown. Here, we demonstrate that transgenic targeting of OGG1 to mitochondria confers significant protection from diet-induced obesity, insulin resistance, and adipose tissue inflammation. These favorable metabolic phenotypes are mediated by an increase in whole body energy expenditure driven by specific metabolic adaptations, including increased mitochondrial respiration in white adipose tissue of OGG1 transgenic (*Ogg1*^*Tg*^) animals. These data demonstrate a critical role for a DNA repair protein in modulating mitochondrial energetics and whole-body energy balance.

## Introduction

Endogenous metabolic byproducts and exogenous oxidants can cause extensive oxidative damage to both genomic and mitochondrial DNA (mtDNA). Unrepaired oxidative DNA damage can in turn lead to mutagenesis, cellular transformation and dysfunction, and cell death^1–4^. The cell therefore has multiple mechanisms to guard against DNA damage and to repair existing damage. The primary pathway for repair of non-bulky oxidatively-induced lesions is the base excision repair (BER) pathway that is initiated by DNA glycosylases. These enzymes, including 8-oxoguanine DNA glycosylase (OGG1), Nei endonuclease VIII-like (NEIL)1, NEIL2, NEIL3, and endonuclease III-like 1 (NTH1), have distinct tissue distributions and substrate specificities to recognize and excise specific subsets of lesions, and in some cases, can further process the damaged site to a single-strand break via an intrinsic AP lyase activity^5–7^.

OGG1 recognizes and cleaves the most common oxidatively-induced DNA lesion, 8-oxo-7,8-dihydroguanine (8-oxoG)^1^. OGG1 localizes to both the nucleus and mitochondria^8–12^ and has been investigated for its role in many disease pathways, including various cancers, and neurological diseases such as Parkinson’s and Alzheimer’s disease^4,5,13–27^. Additionally, we previously reported that mice lacking OGG1 (*Ogg1*^−/−^) are prone to features of metabolic syndrome, including increased body weight and adiposity, fatty liver, elevated triglycerides, skeletal muscle lipid deposition, and impaired glucose tolerance^28,29^. Concomitantly, several groups have reported a correlation between polymorphisms in the *Ogg1* gene and incidence of obesity and type II diabetes in human cohorts^30,31^.

Given the known mitochondrial localization of OGG1 and the importance of mitochondrial metabolism to energy balance, we were interested in determining the relative contribution of mitochondrial OGG1 to body weight regulation. To do this, we utilized a transgenic animal constitutively overexpressing human OGG1-1a targeted to the mitochondria. Here we show that targeting OGG1 localization to mitochondria alone confers significant protection against diet-induced obesity and adiposity through specific metabolic adaptations in epididymal white adipose tissue (eWAT). These data demonstrate, for the first time, a role for a mtDNA repair protein in the maintenance of adipose tissue mitochondrial function and whole-body energy balance. These results also indicate that manipulation of mitochondrial energetics in adipose tissue, specifically through targeting of mtDNA repair, may be a novel and viable approach to manage metabolic disease.

## Results

### Overexpression of mitochondrially-targeted OGG1 significantly protects mice from diet-induced obesity

Given our observations of increased propensity to diet-induced obesity and insulin resistance in *Ogg1*^−/−^ mice^28,29^, especially under conditions of increased oxidative stress, we were interested in determining the relative contribution of mitochondrial OGG1 to whole body energy homeostasis. Therefore, we obtained transgenic animals constitutively overexpressing human OGG1 in the mitochondria (*Ogg1*^*Tg*^) and backcrossed them into a C57BL6J background for over six generations. The initial generation of these mice and confirmation of a selective increase in mtDNA repair activity has been previously reported^32,33^. These mice overexpress the human OGG1-1a isoform that has been targeted for mitochondrial localization via the use of a mitochondrial targeting sequence derived from the *MnSOD* gene. These mice have been shown to have a specific increase in mitochondrial OGG1 activity without any changes in nuclear OGG1 activity levels^32,33^. However, there have been no reports on whole body metabolism, physiology, or pathology in these animals. These *Ogg1*^*Tg*^ mice represent a model of enhanced mitochondrial OGG1 expression and allow us to interrogate the specific role of the mitochondrial form of this DNA repair enzyme in regulating body weight and energy balance.

**Figure 1:**
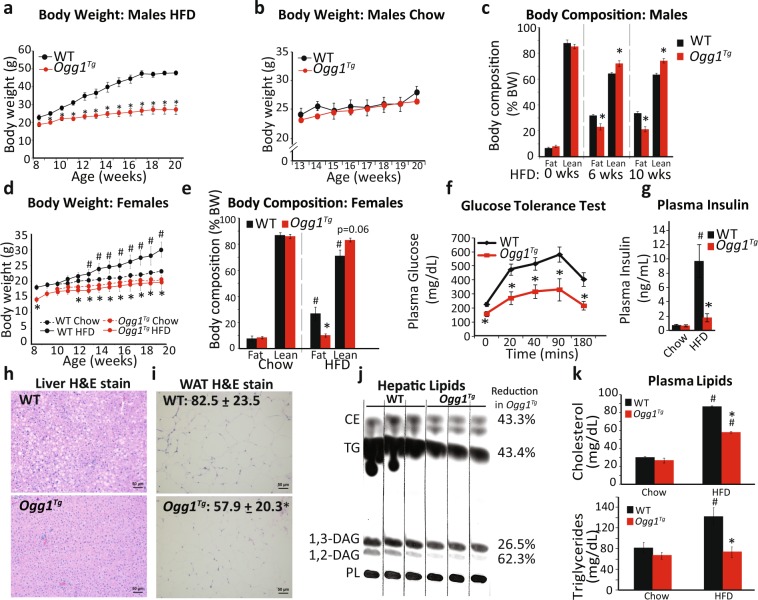
Mitochondrial *OGG1* overexpression confers a favorable metabolic phenotype. The metabolic phenotype of *Ogg1*^*Tg*^ mice was determined. Mice were maintained on standard chow or 60% HFD for twelve weeks, from eight to twenty weeks of age (n = 6 in each cohort). Male mice were utilized except in (**d**,**e**). Body weights were measured weekly (**a**,**b**,**d**), and body composition was determined by NMR (**c**,**e**) at the indicated intervals. Glucose tolerance (**f**) was assessed after seven weeks of HFD feeding. Plasma insulin (**g**) was measured in twenty-week old mice that had been maintained on chow or fed a HFD for twelve weeks. Liver sections (**h**) and eWAT sections (**i**) from HFD-fed mice were stained with H&E to visualize lipid droplets and adipocyte size. Average adipocyte diameter, indicated at the top left of the section, was determined by counting 50 cells per genotype using ImageJ software (**i**). Scale bars (**h**,**i**) represent 50 μM. Hepatic lipids were extracted from livers of HFD-fed animals and separated by thin-layer chromatography, and bands corresponding to CE, TG, and DAG were quantified by densitometric evaluation in Image J (**j**). Plasma triglycerides and cholesterol (**k**) were measured using commercially available kits. Data are expressed as average ± SEM. *p < 0.05 vs. WT mice; ^#^p < 0.05 vs. chow-fed mice. CE, cholesterol esters, DAG, diacylglycerols, eWAT, epididymal white adipose tissue, PL, phospholipids TG, triglycerides.

To determine if enhanced mitochondrial OGG1 expression is protective against diet-induced obesity, age-matched male WT and *Ogg1*^*Tg*^ mice were placed on a high-fat diet (HFD) for 12 weeks. Enhanced mtOGG1 localization resulted in significantly reduced HFD-induced weight gain in male *Ogg1*^*Tg*^ mice (Fig. 1a). Chow-fed mice had similar body weights as WT controls (Fig. 1b). The protection from HFD-induced weight gain was accompanied by a significantly lower fat composition (Fig. 1c) and reduced weights of epididymal fat pads (eWAT) (2.6 ± 0.18 g in WT vs. 1.2 ± 0.26 g in *Ogg1*^*Tg*^, *p* = *0.002*), indicating a role for mitochondrial OGG1 in whole body energy and fat balance. Similar to male animals, female *Ogg1*^*Tg*^ mice also had significantly reduced HFD-induced weight gain and adiposity (Fig. 1d,e), relative to WT counterparts. All further studies were conducted in male mice. Commensurate with the protection from diet-induced adiposity, *Ogg1*^*Tg*^ mice had significantly improved glucose tolerance following injection of a glucose bolus (Fig. 1f). This was accompanied by reduced plasma insulin levels (Fig. 1g), indicating improved insulin sensitivity in these mice. After 12 weeks of HFD feeding, hepatic lipid accumulation was significantly reduced in *Ogg1*^*Tg*^ mice (Fig. 1h,j), indicating that the reduction in fat mass in these mice does not result in lipodystrophic lipid accumulation in non-adipose tissue. Histological evaluation of eWAT revealed smaller adipocytes in *Ogg1*^*Tg*^ epididymal fat depots (Fig. 1i), commensurate with their lean metabolic phenotype. Plasma indices of lipid metabolism (Fig. 1k), including circulating cholesterol and triglycerides were significantly lower in HFD-fed *Ogg1*^*Tg*^ mice, relative to WT counterparts.

### Reduction in body weight is mediated by increased energy expenditure in *Ogg1*^*Tg*^ mice

**Figure 2:**
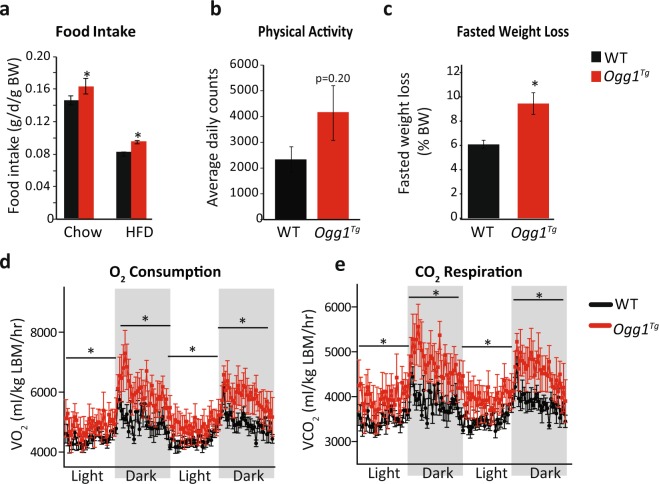
Energy expenditure is increased in *Ogg1*^*Tg*^ mice. *Ad libitum* food intake was measured weekly (**a**) and voluntary physical activity (**b**) within a home-cage setting was determined in chow-fed mice by counting beam breaks over a period of 3 days, after 3 days of acclimatization. Fasted weight loss (**c**) was calculated by weighing mice before and after an overnight (18 hr) fast after 8 weeks of HFD-feeding. O_2_ consumption (**d**) and CO_2_ respiration (**e**) were determined by indirect calorimetry after 10 weeks of HFD feeding over 2 consecutive dark and light cycles, following 2 days of acclimatization.

Food intake was slightly but significantly increased in both chow-fed and HFD-fed *Ogg1*^*Tg*^ mice (Fig. 2a), indicating that their lean phenotype is not mediated by a reduction in food intake. Voluntary physical activity was not significantly altered in *Ogg1*^*Tg*^ mice (Fig. 2b) but tended to be increased, suggesting potential differences in physical activity that may be secondary to the lean phenotype of these animals. Following an overnight fast, *Ogg1*^*Tg*^ mice lost significantly more body weight than WT counterparts (Fig. 2c), suggesting an increase in metabolic rate in these mice. Indeed, indirect calorimetry revealed that both O_2_ consumption and CO_2_ respiration were significantly increased across dark and light cycles (Fig. 2d,e). These data indicate a significant increase in energy expenditure that is consistent with the obesity resistance of *Ogg1*^*Tg*^ mice. Respiratory exchange ratios (VCO_2_/VO_2_) were not different between genotypes (not shown).

### Altered metabolic health of eWAT

**Figure 3:**
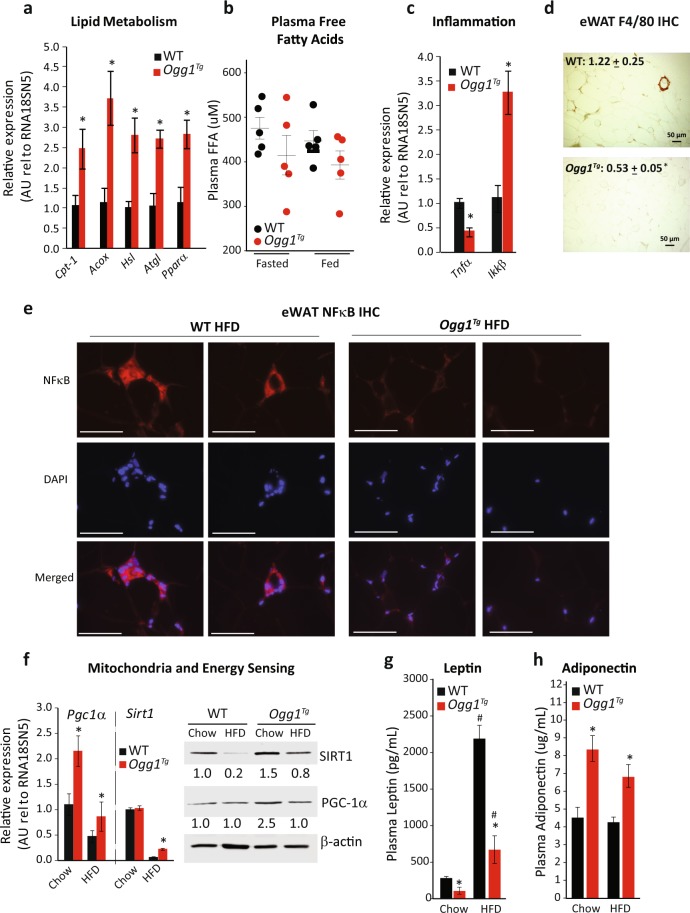
Adipose tissue metabolism is altered in *Ogg1*^*Tg*^ mice. Metabolic parameters were determined in eWAT of WT and *Ogg1*^*Tg*^ mice (n = 6, unless otherwise indicated). Expression of genes regulating lipid catabolism (**a**), inflammation (**c**), and mitochondrial energy metabolism (**f**) was quantitated by qRT-PCR using gene-specific primers. Plasma FFA were determined in HFD-fed and overnight-fasted mice (n = 5) using a colorimetric kit (**b**). Macrophage infiltration into eWAT sections was determined by immunohistochemistry using an antibody against the macrophage protein F4/80, and staining intensity, quantified in ImageJ, is shown in the top left corner of images (**d**). In addition, protein levels of the inflammatory protein NFκB was determined by IHC using an antibody against NFκB, followed by counterstaining with DAPI (**e**). Protein levels of SIRT-1 and PGC-1α were determined by SDS-PAGE (**f**), and β-actin was utilized as a loading control. Plasma leptin (**g**) and adiponectin (**h**) levels were determined by ELISA. Data are expressed as average ± SEM. *p < 0.05 vs. WT mice; ^#^p < 0.05 vs. chow-fed mice. Acox, acyl-CoA oxidase, Atgl, adipose triglyceride lipase, Cpt-1, carnitine palmitoyl transferase-1, eWAT, epididymal white adipose tissue, FFA, free fatty acids, Hsl, hormone-sensitive lipase, Pgc-1α, PPAR-gamma coactivator-1 alpha, Pparα, peroxisome proliferator activated receptor-alpha, Sirt1, sirtuin 1.

Given these changes in adiposity and energy expenditure, as well as the observation of smaller cell size in eWAT, we measured markers of the metabolic health of eWAT from WT and *Ogg1*^*Tg*^ mice. Commensurate with their reduced adipose mass, several genes regulating fatty acid oxidation, including *Cpt-1*, *Acox*, *Hsl*, *Atgl*, and *Ppar-α* were all significantly upregulated in eWAT from *Ogg1*^*Tg*^ mice, relative to WT counterparts (Fig. 3a). The increase in *Hsl* and *Atgl* levels indicate an increase in lipolytic processes in eWAT of *Ogg1*^*Tg*^ animals. As these increases in lipolytic markers were accompanied by increased expression of fatty acid oxidation genes, we did not observe any elevation in steady-state plasma free fatty acid levels in *Ogg1*^*Tg*^ mice, under either HFD-fed or overnight-fasted conditions (Fig. 3b). Interestingly, fatty acid oxidation genes were elevated only in eWAT of HFD-fed mice, but not in other metabolically active tissues such as liver, brown adipose tissue, or skeletal muscle (Supplementary Fig. S1a), indicating that local oxidation of mobilized fatty acids in eWAT may constitute a futile energy cycle and contribute to the observed lean phenotype of these mice. The significance of metabolic changes being localized to adipose tissue is discussed in greater detail below.

Obesity is associated with increased inflammation in adipose tissue^34–38^. Expression of the pro-inflammatory cytokine tumor necrosis factor alpha (*Tnfα*) was reduced and expression of inhibitor of NF-κB (*Ikk-β*), was robustly induced in *Ogg1*^*Tg*^ eWAT, indicating reduced levels of inflammation in eWAT of *Ogg1*^*Tg*^ mice (Fig. 3c). We confirmed these results via staining for a marker of macrophage infiltration in eWAT sections. The appearance of F4/80-positive crown-like structures was significantly reduced in eWAT of HFD-fed *Ogg1*^*Tg*^ mice (Fig. 3d), indicating a decrease in metabolically deleterious adipose inflammation in these mice. Additionally, staining for the pro-inflammatory nuclear factor-kappa B (NF-κB) along with DAPI nuclear co-staining revealed a reduction in overall cellular NF-κB levels in eWAT of HFD-fed *Ogg1*^*Tg*^ mice and a decrease in nuclear and peri-nuclear localization of NF-κB. These results are consistent with reduced inflammation in eWAT of *Ogg1*^*Tg*^ animals (Fig. 3e).

### Increased mitochondrial metabolism in eWAT of *Ogg1*^*Tg*^ mice

Mitochondrial biogenesis and key mitochondrial functions such as fatty acid oxidation are known to be under the transcriptional control of the transcriptional co-activator peroxisome proliferator-activated receptor gamma coactivator 1-alpha (PGC-1α). Expression of PGC-1α was significantly increased in eWAT from *Ogg1*^*Tg*^ mice, especially under chow-fed conditions (Fig. 3f). Notably, we previously reported that mice lacking OGG1 (*Ogg1*^−/−^) are prone to diet-induced obesity^28^, which was accompanied by a significant reduction in *Pgc-1α* expression. Therefore, these findings of enhanced PGC-1α expression in mice overexpressing mitochondrial OGG1 (Fig. 3f) provide further evidence for a role for PGC-1α in mediating metabolic phenotypes downstream of OGG1 levels. This increase in PGC-1α was apparent only in eWAT of *Ogg1*^*Tg*^ mice; tissues such as BAT, heart, liver, and muscle from *Ogg1*^*Tg*^ mice did not show a similar induction (Supplemental Fig. S1b).

PGC-1α undergoes post-translational, as well as transcriptional regulation and activation by the histone deacetylase, Sirtuin1 (SIRT1). Transgenic overexpression of SIRT1 increases energy expenditure and confers protection against obesity^39^. Interestingly, we found that while HFD-feeding reduced SIRT1 gene and protein levels, this reduction was greater in WT mice than in *Ogg1*^*Tg*^ mice. *Ogg1*^*Tg*^ animals retained higher SIRT1 expression in eWAT (Fig. 3f). Similar to PGC-1α, the increase in *Sirt1* gene expression seen in eWAT was not observed in other tissues of *Ogg1*^*Tg*^ mice (Supplemental Fig. S1a).

In addition to being a site of lipid storage and active lipid remodeling, adipose tissue is also a source of important cytokines such as leptin and adiponectin. Since these adipokines are critical to maintaining metabolic health, we measured levels of circulating leptin and adiponectin in WT and *Ogg1*^*Tg*^ mice (Fig. 3g,h). Plasma leptin levels were correlated with adipose mass such that while HFD-feeding increased leptin levels in all animals, *Ogg1*^*Tg*^ mice had significantly lower plasma leptin under both dietary conditions (Fig. 3g). Plasma adiponectin was unchanged by HFD-feeding in either genotype. Interestingly, however, *Ogg1*^*Tg*^ mice had significantly higher plasma adiponectin than WT counterparts under both dietary conditions (Fig. 3h). Concomitantly, adiponectin gene expression was significantly increased in HFD-fed *Ogg1*^*Tg*^ mice, relative to WT counterparts (relative gene expression of 1.2 ± 0.3 in WT mice vs. 4.6 ± 0.6. *Ogg1*^*Tg*^ animals, *p* = *0.002*) Although produced by adipocytes, plasma adiponectin is known to be inversely correlated with body weight and directly proportional to insulin sensitivity. Importantly, SIRT1 is a known regulator of adiponectin expression. Therefore, the increase in both SIRT1 levels (Fig. 3f), as well as adiponectin expression and plasma adiponectin levels (Fig. 3h) indicate that mitochondrial OGG1 may result in activation of the SIRT1-adiponectin axis. This in turn could serve to upregulate PGC-1α levels, resulting in the increased energy expenditure and the lean metabolic phenotype observed in *Ogg1*^*Tg*^ mice. Future studies are focused on delineating the importance of the PGC-1α -SIRT1 axis to the observed metabolic phenotype of *Ogg1*^*Tg*^ mice.

### Mitochondrial protein content and respiration are increased in eWAT of *Ogg1*^*Tg*^ mice

**Figure 4:**
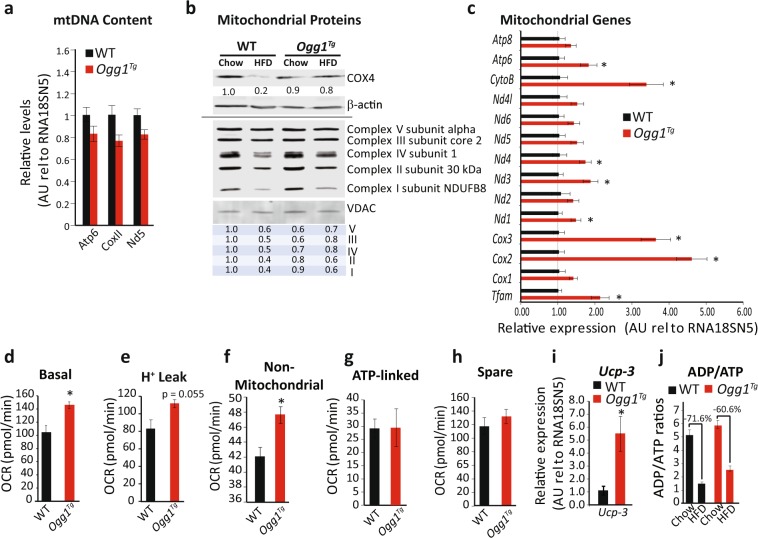
Mitochondrial content and function are altered in eWAT of *Ogg1*^*Tg*^ mice. Mitochondrial content and function were evaluated in eWAT of WT and *Ogg1*^*Tg*^ mice (n = 6, unless otherwise indicated). mtDNA (**a**) and protein (**b**) content were determined by qRT-PCR and SDS-PAGE after 12 weeks of HFD, respectively. Expression of OXPHOS proteins (**b**) was quantified using the program ImageStudio Lite. Expression of mitochondrial genes and *Tfam* (**c**), as well as *Ucp-3* (**i**) was measured by qRT-PCR in eWAT from HFD-fed mice. Mitochondrial respiration (**d**), proton leak (**e**), non-mitochondrial respiration (**f**), ATP-linked respiration (**g**), and spare capacity (**h**) were measured using a Seahorse Biosciences Extracellular Flux Analyzer and 10 adipose punches from 4 chow-fed animals per genotype (40 replicates per genotype). ADP/ATP ratio was quantified using a fluorometric kit (**j**). Data are expressed as average ± SEM. *p < 0.05 vs. WT mice. Atp, ATP Synthase, Cox, cytochrome c oxidase, CytoB, cytochrome B, eWAT, epididymal white adipose tissue, Nd, NADH dehydrogenase, Nd4l, NADH:Ubiquinone Oxidoreductase Core Subunit 4L, OCR, O2 consumption rate, OXPHOS, oxidative phosphorylation, Tfam, mitochondrial transcription factor A, Ucp3, uncoupling protein 3, VDAC, voltage-dependent anion channel.

mtDNA content was not significantly changed in *Ogg1*^*Tg*^ animals (Fig. 4a). Protein levels of cytochrome C oxidase subunit 4 (COX4), the terminal enzyme of the mitochondrial respiratory chain and an indicator of mitochondrial content, were reduced by HFD-feeding in WT mice (Fig. 4b). However, *Ogg1*^*Tg*^ mice did not show a similar downregulation of COX4, indicating preservation of mitochondrial content in eWAT of these mice, even upon consumption of a hypercaloric diet (Fig. 4b). Similarly, levels of several subunits of the oxidative phosphorylation chain, including complexes I, II, and IV were significantly reduced by HFD-feeding in mitochondrial extracts of WT mice. However, a similar reduction was not evident in extracts from eWAT of *Ogg1*^*Tg*^ mice (Fig. 4b). This preservation of mitochondrial content after HFD-feeding led us to speculate that transcription of mitochondrially-encoded genes may be increased in *Ogg1*^*Tg*^ mice. To test this hypothesis, we measured expression of mitochondrial transcription factor A (*Tfam)*, a key regulator of mitochondrial transcription, as well as expression of all thirteen mitochondrially-encoded genes (Fig. 4c) in eWAT of HFD-fed mice. Indeed, levels of *Tfam*, as well as several mitochondrial transcripts were significantly upregulated in *Ogg1*^*Tg*^ mice, indicating increased transcription of mitochondrially-encoded genes in these animals (Fig. 4c).

To determine if the observed changes in mitochondrial genes and proteins translate to alterations in mitochondrial function, we measured O_2_ consumption in eWAT punches from chow-fed mice using a Seahorse Biosciences analyzer. Basal O_2_ consumption was significantly increased in eWAT from *Ogg1*^*Tg*^ mice (Fig. 4d), relative to WT controls. Upon suppression of ATP-linked O_2_ consumption by the addition of oligomycin, it was apparent that a large part of the increase in basal respiration was attributable to non-ATP-linked respiration, stemming from proton leak in *Ogg1*^*Tg*^ eWAT (Fig. 4e). In addition to proton leak, non-mitochondrial respiration was significantly higher in eWAT of *Ogg1*^*Tg*^ animals (Fig. 4f). ATP-linked (Fig. 4g) and spare respiratory capacity (Fig. 4h) were not different between WT and *Ogg1*^*Tg*^ mice. To further confirm the apparent increases in proton leak (Fig. 4e), we measured expression of uncoupling proteins 1–3 in WAT. *Ucp-1* levels were not significantly induced in eWAT of *Ogg1*^*Tg*^ mice (relative expression WT: 1.04 ± 0.20 vs. *Ogg1*^*Tg*^: 0.73 ± 0.20). *Ucp-2* levels were also unchanged (WT: 1.13 ± 0.29 vs. *Ogg1*^*Tg*^: 0.96 ± 0.21). Interestingly, expression of *Ucp*-3 was robustly upregulated in eWAT of *Ogg1*^*Tg*^ animals, relative to WT controls (Fig. 4i). While the precise function of UCP-3 is still under debate, increased *Ucp-3* expression has been reported to increase uncoupling and fatty acid oxidation, that combine to protect mice from diet-induced obesity^40–43^. Thus, our observation of increased *Ucp-3* expression in eWAT of *Ogg1*^*Tg*^ mice (Fig. 4i) is consistent with their increased proton leak (Fig. 4e) and higher expression of fat oxidation genes (Fig. 3a), as well as with their resistance to diet-induced obesity (Fig. 1).

Taken together, these data indicate that while O_2_ consumption is higher in eWAT from *Ogg1*^*Tg*^ animals, it is not coupled to ATP production and potentially results in a futile cycle of energy dissipation via increased proton leak. Given these indications of increased uncoupled respiration in adipose tissue from *Ogg1*^*Tg*^ mice, we measured ADP/ATP ratios in these tissues. HFD-feeding reduced ADP/ATP ratios in eWAT of both WT and *Ogg1*^*Tg*^ mice, which is consistent with the more energy-replete state of adipose tissue after prolonged HFD-consumption (Fig. 4j). Interestingly, however, the reduction in the ADP/ATP ratio upon HFD-feeding was smaller in *Ogg1*^*Tg*^ mice, relative to WT animals (60.6% vs. 71.6%, respectively). These data suggest that despite HFD-feeding, eWAT from *Ogg1*^*Tg*^ mice may be less energy replete than eWAT of WT counterparts, consistent with the observed increases in proton leak (Fig. 4e) and *Ucp-3* expression (Fig. 4i).

### Mitochondria are elongated in eWAT of *Ogg1*^*Tg*^ mice

**Figure 5:**
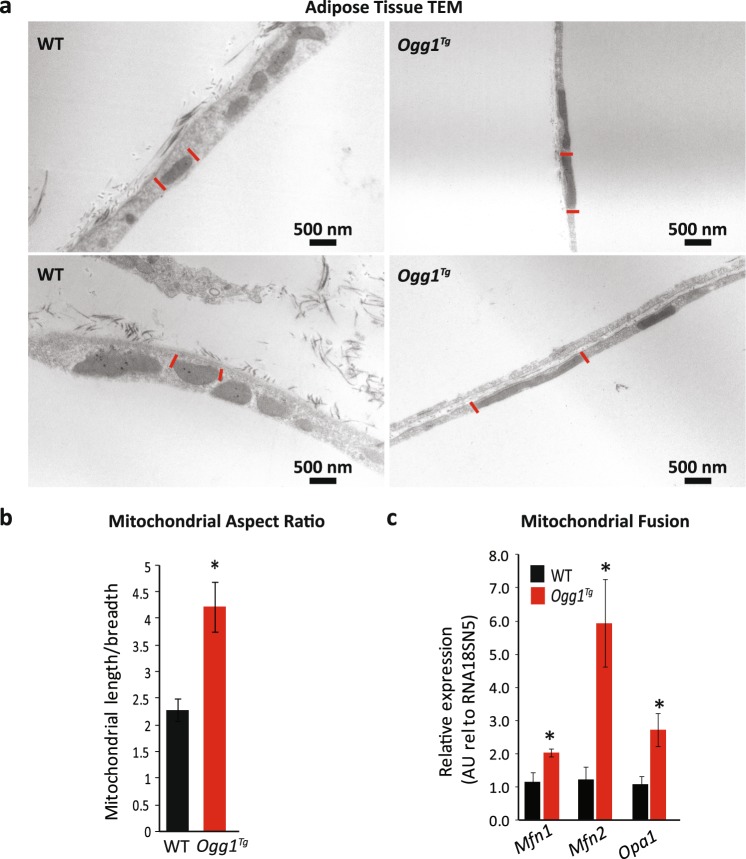
Mitochondria are elongated in eWAT of *Ogg1*^*Tg*^ mice. Mitochondrial ultrastructure and dynamics were evaluated in eWAT from HFD-fed WT and *Ogg1*^*Tg*^ mice (n = 6). Mitochondria were visualized by TEM (**a**); red lines are used to identify boundaries of one representative mitochondrion in each image. Mitochondrial length and breadth were measured across 20 mitochondria from 6 mice to determine average aspect ratio (**b**). Expression of genes regulating mitochondrial dynamics was quantitated by qRT-PCR in eWAT of HFD-fed mice (**c**). Scale bar (**a**) represents 500 nM. Data are expressed as average ± SEM. *p < 0.05 vs. WT mice. eWAT, epididymal adipose, Mfn, mitofusin, Opa1, mitochondrial dynamin-like GTPase.

Apart from mitochondrial content and protein levels, mitochondrial ultrastructure is an important determinant of function. For instance, an energy-replete or energy-dense cell is thought to contain cells with more fragmented mitochondria, while an energy-poor cell tends to retain mitochondria in their elongated state to assist with energy production^44^. To determine if mitochondrial OGG1 results in alterations in mitochondrial structure, adipose sections from HFD-fed WT and *Ogg1*^*Tg*^ mice were fixed and visualized by TEM. Interestingly, mitochondria from *Ogg1*^*Tg*^ mice were increased in length and were more electron dense than mitochondria from WT mice (Fig. 5a). Mitochondrial aspect ratio, calculated as average mitochondrial length/breadth, was significantly higher in eWAT from *Ogg1*^*Tg*^ mice, relative to WT controls (Fig. 5b). Concomitantly, eWAT from *Ogg1*^*Tg*^ mice also had higher expression of several key genes that regulate mitochondrial fusion (Fig. 5c), including the mitofusin genes (*Mfn-1*, *Mfn-2*) and mitochondrial dynamin-like GTPase (*Opa-1*). A similar increase in *Mfn* levels and mitochondrial fusion was previously reported in a cell culture model of cardiomyocytes transduced with adenoviral mitochondrial OGG1^45^. Conversely, we have previously reported that OGG1-deficient (*Ogg1*^−/−^) mice have markers of increased mitochondrial fission and fragmentation^29^, indicating that OGG1 levels, and particularly mitochondrial OGG1 levels, may serve to regulate cellular mitochondrial dynamics. The observed increases in mitochondrial fusion in eWAT of *Ogg1*^*Tg*^ mice may be secondary to their apparent inability to couple ATP production to oxidative phosphorylation, as suggested by the increase in *Ucp-3* levels (Fig. 4i) and mitochondrial proton leak (Fig. 4e) in these animals. The observation of increased mitochondrial length in eWAT of these mice is consistent with their lean metabolic phenotype.

### Role for 8-oxoG in mediating metabolic phenotypes

**Figure 6:**
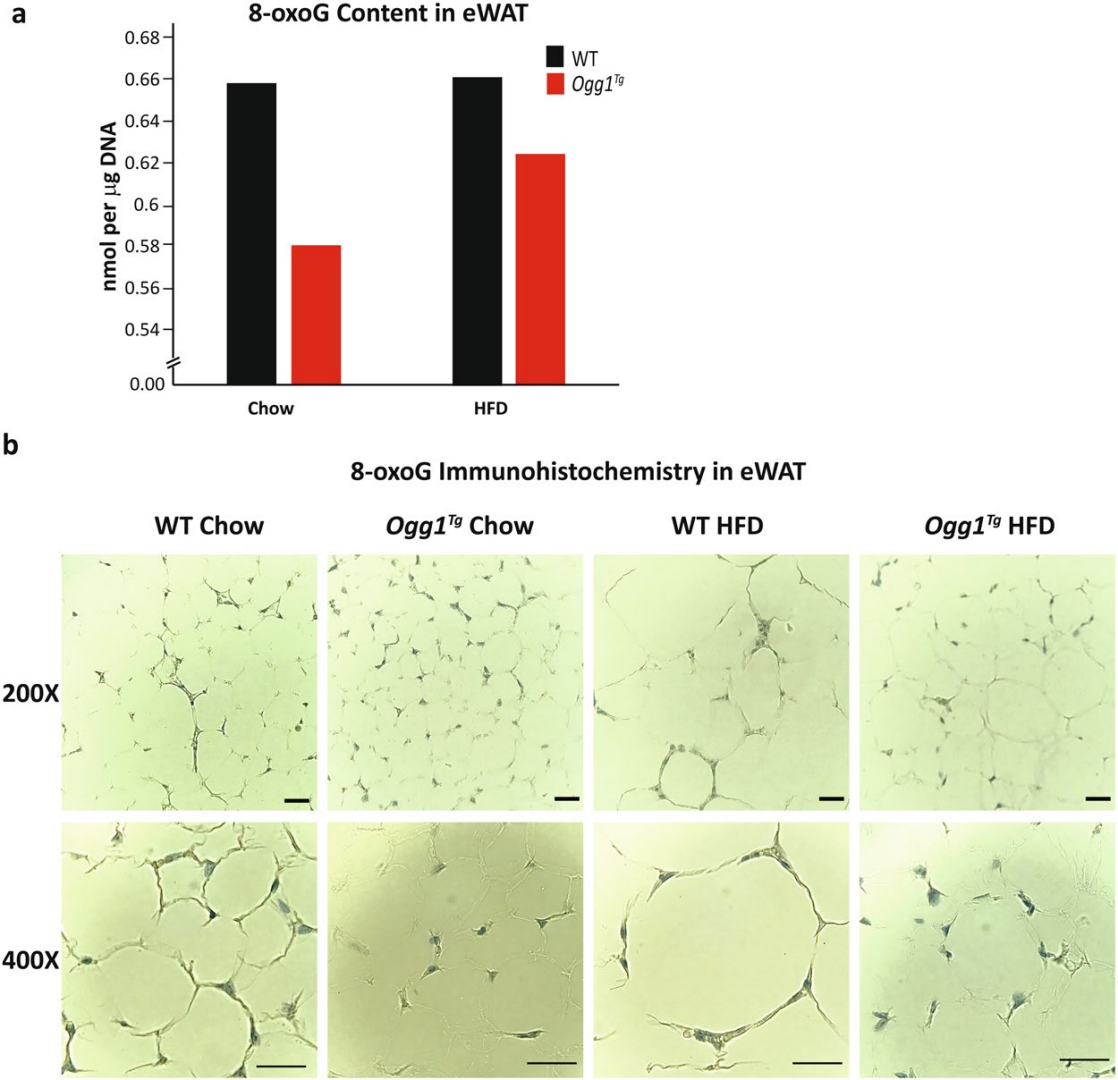
8-oxoG in eWAT. Adipose tissue 8-oxoG content was determined by ELISA (**a**) using DNA from eWAT pooled from 6–8 animals per cohort. Immunohistochemical detection of 8-oxoG (**b**) was performed using an anti-8oxoG antibody, followed by hematoxylin counterstaining. Images are representative of 6 animals per cohort. Scale bars (**b**) represent 50 μM.

In order to determine if these changes in metabolic efficiency in eWAT of *Ogg1*^*Tg*^ mice are correlated with changes in tissue 8-oxoG content, we measured 8-oxoG levels in eWAT of both chow-fed and HFD-fed mice. eWAT from all mice in a cohort (n = 6–8) were pooled, total DNA was isolated, and 8-oxoG content was determined by ELISA. We observed a trend for reduced 8-oxoG levels in eWAT of *Ogg1*^*Tg*^ mice, under both chow- and HFD-fed conditions (Fig. 6a). To further quantify tissue 8-oxoG content, eWAT sections were stained with an antibody against 8-oxoG and examined by microscopy. eWAT from both chow- and HFD-fed *Ogg1*^*Tg*^ mice had decreased intensity of 8-oxoG staining, relative to WT counterparts (Fig. 6b). Additional images and negative and positive controls for 8-oxoG immunohistochemistry are presented in Supplementary Fig. S2. These data are suggestive of a role for tissue 8-oxoG content in mediating at least some of the metabolic phenotypes observed in eWAT of *Ogg1*^*Tg*^ mice. However, additional roles beyond 8-oxoG repair have been ascribed to OGG1, and the potential significance of these functions of OGG1 to metabolic regulation cannot be ruled out and are discussed below.

### Mitochondrial OGG1 attenuates weight gain in *Ogg1*^−/−^ mice

**Figure 7:**
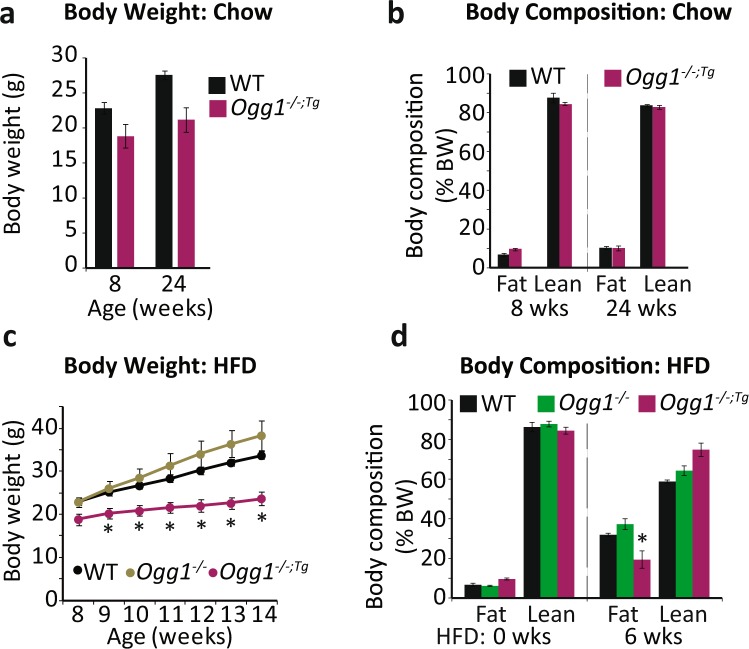
Mitochondrial OGG1 overexpression attenuates diet-induced weight gain in *Ogg1*^−/−^ *mice.* *Ogg1*^*Tg*^ mice were crossed with *Ogg1*^−/−^ mice (n = 6) and either maintained on chow diet or transitioned to a high-fat diet (HFD) at 8 weeks of age. Chow-fed mice were weighed (**a**) and body composition was determined (**b**) at the indicated intervals. HFD-induced weight gain (**c**) was determined weekly, and body composition was measured by NMR (**d**) before and 6 weeks after the start of HFD-feeding. *p < 0.05 vs. WT and *Ogg1*^−/−^.

All the studies described above were carried out in mice with transgenic overexpression of mitochondrial OGG1 in the context of intact nuclear and mitochondrial mouse OGG1. To determine if mitochondrial OGG1 alone is sufficient to confer these favorable metabolic phenotypes, we bred *Ogg1*^*Tg*^ mice to *Ogg1*^−/−^ animals that lack any endogenous OGG1 and placed age-matched WT, *Ogg1*^−/−^, and *Ogg1*^−/−*;Tg*^ animals on chow or HFD. While there were no significant differences in body weight (Fig. 7a) and composition (Fig. 7b) in chow-fed mice, *Ogg1*^−/−*;Tg*^ animals were remarkably protected from both HFD-induced weight gain (Fig. 7c) and adiposity (Fig. 7d). These results indicate that nuclear OGG1 is dispensable to the role of OGG1 as a modulator of energy metabolism and that mitochondrially localized OGG1 is sufficient to confer a lean metabolic phenotype.

## Discussion

By utilizing a model of OGG1 expression targeted to mitochondria, we demonstrate here that mitochondrial OGG1 makes an important contribution to whole body energy balance. Indeed, even in the absence of any endogenous OGG1, as in the *Ogg1*^−/−*;Tg*^ animals, mitochondrially-targeted OGG1 significantly protects against development of obesity. The protection from obesity in *Ogg1*^*Tg*^ mice is a result of an increase in overall energy expenditure stemming from increases in mitochondrial respiration and uncoupling specifically in eWAT. Concomitantly, *Ogg1*^*Tg*^ mice also display significant alterations in mitochondrial structure and protein content in eWAT, indicating an important role both for OGG1 in modulating adipose function, as well as for adipose tissue mitochondrial metabolism in regulating whole body energy homeostasis. This is the first report of metabolic protection in an animal model with transgenic targeting of a mtDNA repair protein. Our prior reports of obesity-proneness in OGG1-deficient animals^28,29^, combined with these data, cement a role for OGG1 in the regulation of metabolic homeostasis.

In human cells, eight different isoforms of *OGG1* mRNA (*OGG1-1a, -1b, -1c, -2a, -2b, -2c, -2d* and *-2e*), and therefore potentially proteins, can be generated from the *OGG1* gene. They all share an N-terminal mitochondrial-targeting sequence (MTS). However, the human *OGG1-1a* isoform, which was utilized in the generation of the transgenic mice reported here, is the only one to contain a C-terminal nuclear localization signal^10,46–48^. Indeed, at the time of generation of these transgenic mice, the -1a isoform considered a *de facto* nuclear OGG1 with robust 8-oxoG excision activity. However, a recent report has indicated that the *hOGG1-1a* isoform can be localized to both the nucleus and mitochondria and is capable of repairing 8-oxoG in both compartments^47^. Another group previously reported a metabolic phenotype in a distinct transgenic line with mitochondrial targeting of OGG1^49^. However, this transgenic animal was generated via targeted overexpression of the *hOGG1-2a* (or *β*) isoform and had a reported increase in body weight, secondary to significant hyperphagia^49^. In contrast, the transgenic mice used in the present study, with *hOGG1-1a* overexpression, did not exhibit hyperphagia and were remarkably protected from diet-induced obesity (Fig. 1). These discrepancies are very likely attributable to the choice of OGG1 isoform in these two transgenic models. Notably, the 2a isoform has been reported to be devoid of glycosylase activity and to function primarily via a physical interaction with mitochondrial aconitase to prevent the latter’s oxidative modification^50^. Conversely, the −1a isoform has been demonstrated to have robust glycosylase activity and has been shown in multiple cellular models to be protective against oxidant-induced injury^32,51–55^.

In light of these striking metabolic phenotypes upon mtOGG1 targeting, a question that remains to be answered is whether these changes are related to the role of OGG1 in 8-oxoG repair or whether these changes are attributable to other known or as yet unidentified functions of OGG1. Our findings of a trend for reduced 8-oxoG levels in both chow-fed and HFD-fed *Ogg1*^*Tg*^ mice (Fig. 6, Supplementary Fig. 2) are suggestive of a role for 8-oxoG repair in mediating at least some of the metabolic phenotypes observed. It has been suggested that OGG1 and 8-oxoG levels may play an important role in modulating global transcriptional responses. Despite the vulnerability of 8-oxoG to pre-mutagenic oxidation, promoter regions of the majority of transcribed genes are enriched in guanines, leading to the speculation that guanine oxidation may serve as a mechanism to modulate gene expression in response to acute oxidative challenges^56,57^. Indeed, 8-oxoG levels are about five-fold higher in transcriptionally active euchromatin than in transcriptionally-silent heterochromatin areas^58^. Further, under cellular stress conditions, such as following hypoxia, a specific increase in 8-oxoG formation and recruitment of OGG1 to promoter areas is required for upregulation of hypoxia- responsive genes such as VEGF^59^. OGG1 activity has also been shown to be important to DNA methylation, as oxidation of guanines in CpG sequences renders DNA a poorer substrate for DNA methyltransferases^60–62^. Furthermore, demethylation of histones generates local increases in H_2_O_2_ levels, recruiting OGG1 to sites of active demethylation, further implicating this DNA repair protein in modulating transcription^63^. These data are supportive of a role for OGG1 and 8-oxoG in modulating gene expression in response to specific cellular stressors. It is notable that in our model of OGG1 mitochondrial localization, transcription of mitochondrially-encoded genes was upregulated (Fig. 4c), suggesting a role for mitochondrial OGG1 in modulating mitochondrial transcriptional responses. Apart from a role in regulating transcription, OGG1 bound to its substrate 8-oxoG, has been demonstrated to function as a guanine exchange factor stimulating the activity of Ras-GTPases. This functionality of OGG1 has been demonstrated to be important in modulating innate and adaptive inflammatory processes^57,64,65^. Whether this functionality of OGG1 is important in mediating adipose tissue inflammation is yet to be determined. These data regarding functions for OGG1 that go beyond its role in 8-oxoG excision are compelling and must be considered with regard to its function as a metabolic regulator. Ongoing studies will be focused on utilizing DNA-repair deficient mutants of the OGG1 protein to address the question of whether these transcriptional changes and favorable metabolic phenotypes upon mitochondrial OGG1 localization are dependent on or independent of its DNA repair functionality.

Optimal mitochondrial function is essential for the health of all cell types. However, suboptimal mitochondrial function can have varying degrees of impact on the function of different cells and organs. Tissues that rely heavily on mitochondrial function, including oxidative skeletal muscle, heart, and brain, as well as liver and adipose tissue may be expected to be impacted most by alterations in mitochondrial function. In our investigations, several metabolic changes were apparent only in eWAT of *Ogg1*^*Tg*^ mice. For instance, the metabolically favorable increases in *Sirt1* and *Pgc-1α* levels (Fig. 3f) and the measurable increases in mitochondrial genes, proteins, and mitochondrial length (Figs 4 and 5) were evident only in eWAT. Similar changes were not observed in other tissues examined, including liver, BAT, or skeletal muscle (Supplementary Fig. S1). One apparent explanation for this tissue selectivity may lie in the use of a hypercaloric, high-fat diet as the metabolic stressor in these studies. Under conditions of chronic overfeeding, white adipose depots display great plasticity, expanding from about 5% to over 30% of body weight within just 12 weeks of overnutrition. It is plausible that under these conditions, mitochondrial function in adipose tissue is extremely important not only to optimal function of the adipose organ, but also to overall energy metabolism in the whole animal. Ongoing studies in the lab are focused on studying the metabolic consequences of adipose-specific overexpression of OGG1 and the importance of maintaining oxidative metabolism in adipose tissue during periods of proliferation and expansion.

Our findings of increased energy expenditure and protection from diet-induced obesity in *Ogg1*^*Tg*^ mice indicate that a role for DNA damage and repair must be considered when evaluating metabolic health. Furthermore, our data indicate that approaches to enhance mitochondrial respiration in WAT through upregulation of mtOGG1 may represent a novel method to combat adipose tissue hypertrophy and inflammation. Strategies to reduce 8-oxoG levels in adipose tissue could include the use of mild uncouplers or targeted antioxidants to reduce ROS generation, or novel small molecules to specifically enhance mitochondrial OGG1 activity.

## Materials and Methods

### Animals and metabolic phenotyping

The generation of *Ogg1*^*Tg*^ and *Ogg1*^−/−^ mice has been previously described^9,33^. For our studies, transgenic mice were backcrossed at least six generations to the C57BL/6 J background at OHSU and Rutgers University. *Ogg1*^−/−^ mice have been backcrossed to the C57BL/6 J background for over twenty generations. Mice for experimental procedures were produced by mating one *Ogg1*^*Tg*^ parent to a non-transgenic parent to produce experimental and control animals in all litters. Age-matched males were used throughout the studies, except in Fig. 1d,e, where data from female mice are shown. For diet studies, 8-week old mice were individually housed and given *ad libitum* access to rodent chow or a HFD (Research Diets D12492, New Brunswick, NJ; 60% fat, 20% protein, 20% carbohydrate by calories; 5.24 kcal/g metabolizable energy) for 12 weeks. Mice were euthanized by isoflurane overdose followed by exsanguination via cardiac puncture. For *in vivo* procedures, all efforts were made to minimize discomfort and suffering, in accordance with approved animal care protocols. The breeding and care of animals are in accordance with the protocols approved by the Animal Care and Use Committee of Oregon Health & Science University, Portland, Oregon and Rutgers University, New Brunswick, New Jersey. Metabolic phenotyping studies including body weight, food intake, energy expenditure, activity, body composition, lipid analyses, glucose tolerance, and tissue histology were conducted as has been previously described^28^. Briefly, energy expenditure was measured by indirect calorimetry (Oxymax, Columbus Instruments, Columbus, OH) after 10 weeks of feeding. Oxygen consumption (VO_2_) and carbon dioxide production (VCO_2_) were simultaneously determined in individually housed animals with *ad libitum* access to HFD and water. Following a 48-hr acclimation period, VO_2_ and VCO_2_ were recorded for 48 hrs, including two 12-hr dark and 12-hr light phases. Samples were recorded every 2 mins with a room air reference taken every 12 mins. Glucose tolerance was assessed 7 weeks after the start of HFD feeding. Briefly, mice were fasted for 4 hrs followed by i.p. injection of 20% dextrose at a rate of 1 g/kg body weight. Blood was collected at the indicated intervals for assessment of plasma glucose using the glucose oxidase method, as previously described^66^. Plasma insulin (Millipore, St. Charles, MO), plasma leptin (RayBiotech, Norcross, GA) and plasma adiponectin (Abcam, Cambridge, MA) were measured by ELISA. Data regarding metabolic phenotyping are presented from one HFD-feeding study, and HFD-feeding has been repeated three additional times with similar data obtained. For ELISA-based or colorimetric measurements, all analyses were performed in triplicate per animal with an n of 6 animals per cohort, unless otherwise indicated.

### DNA and RNA analyses

mtDNA content was measured by qPCR using primers directed against three different regions of mtDNA, as previously described^66^; data were normalized for the high-copy number nuclear gene, RNA18SN5. RNA for quantitative real-time PCR (qPCR) was isolated using Tri-reagent RT (MRC, Inc., Cincinnati, OH) and the Qiagen RNeasy kit. 1 μg of RNA was reverse-transcribed using the Superscript III first-strand synthesis system (Invitrogen, Carlsbad, CA). qPCR was performed on a QuantStudio 3 Real-Time PCR System (Applied Biosystems) using gene-specific primers. Gene expression was normalized to expression of RNA18SN5. Primer sequences are listed in Table 1. All analyses were performed in triplicate per animal with a sample size of 6 animals per cohort, unless otherwise indicated. For 8-oxoG measurement, total DNA was isolated using the Qiagen DNA isolation kit from eWAT pooled from 6–8 animals per cohort. 8-oxoG was measured by ELISA (Trevigen, Gaithersburg, MD), following the manufacturer’s protocol.

**Table 1:** Primer list.

Gene name	Forward primer (5′ ⋙ 3′)	Reverse primer (5′ ⋙ 3′)
*Atp6*	AAATATTAGCCCACCAACAG	CTAGGAGGGTGAATACGTAG
*Atp8*	AGTCTCATCACAAACATTCCC	GTTAGTGATTTTGGTGAAGGTG
*Cox1*	CCTTTGCTTCAAAACGAGAA	ATAGGTTGGTTCCTCGAATG
*Cox2*	CAAGCAACAGTAACATCAAACC	GTGGAACCATTTCTAGGACAA
*Cox3*	TGTTTGCCTACTACGACAAC	GGAAAAGTCAGACTACGTCTAC
*Cytob*	GTACTGAATCCTAGTAGCCAA	AGTATGAGATGGAGGCTAGT
*Ikkβ*	AACATCGTTCTGCAGCAAGGA	TGCACAGACTGCCCTGAT
*Mfn1*	GGTTGCACCTTTGGAAGTGT	GCCGGTTCCACTGTTTCTAA
*Mfn2*	ATGTTACCACGGAGCTGGAC	AACTGCTTCTCCGTCTGCAT
*Nd1*	CGCCCTAACAACTATTATCTTCC	GAAGCGTGGATAAGATGCTC
*Nd2*	GGCCTTCCACCACTAACAGG	AGGGTGGAAAATATTAGGTTGGGT
*Nd3*	ACAAGCTCTGCACGTCTACC	GCTCATGGTAGTGGAAGTAGAAGAG
*Nd4*	TTAACCTCCAACCCTCACAC	AGGCCTGTAATTAGTTTTGGAC
*Nd4l*	CCATACCAATCCCCATCACC	CGTAATCTGTTCCGTACGTGTT
*Nd5*	ACCCATAAAATCTCTCAACC	GTGGTTATGTTTGTGTGAAG
*Nd6*	AAAACGATCCACCAAACCCT	GGTTAGCATTAAAGCCTTCACC
*Opa1*	CAGCACAATGCTTTGGAAGA	CCTTGAGACGACCTTGAAGC
*Pgc-1α*	TCGATGTGTCGCCTTCTTGC	ACGAGAGCGCATCCTTTGG
*Sirt1*	GTCTCCTGTGGGATTCCTGA	CAAACATGGCTTGAGGGTCT
*Tfam*	GGAAGAGCAGATGGCTGAAG	CCCAATGACAACTCCGTCTT
*Tnfa*	TGGCCTCTCTACCTTGTTGCC	GACAGCCTGGTCACCAAATCAG
*Ucp3*	CTGCACCGCCAGATGAGTTT	ATCATGGCTTGAAATCGGACC

Sequence of forward and reverse primers used in manuscript.

### Protein analyses

Protein levels were determined by SDS-PAGE followed by incubation with commercially available primary antibodies, HRP- or Alexa-fluor conjugated secondary antibodies, and detection using enhanced chemiluminescence or fluorescence imaging, respectively. VDAC, COXIV, and OXPHOS antibodies were all obtained from Abcam (Cambridge, MA) and used at a manufacturer recommended dilution of 1:1000 for Western blotting. For Western blots, membranes were cut using molecular weight standards as guides to allow for blotting of protein of interest and loading controls on the same membrane. For immunohistochemistry, formalin-fixed, paraffin-embedded tissues were cut at a thickness of 5 μM and mounted onto charged glass slides by the OHSU and Rutgers Histopathology Cores. A proteinase K-mediated method was used for antigen retrieval for F4/80 staining; a citrate heating method was used for antigen retrieval for NF-κB IHC; and HCl incubation followed by boric acid quenching was utilized for antigen retrieval for 8-oxoG IHC. Following incubation with primary antibodies against F4/80 (1:500; Serotec; kind gift of Dr. Lisa Coussens, OHSU), NF-κB (1:100, Cell Signaling Technology, Danvers, MA), or 8-oxoG (1:100, Stressmarq Biosciences, Victoria, Canada) slides were incubated with appropriate secondary antibodies and detected using DAB as a substrate and brightfield imaging (F4/80, 8-oxoG) or by direct fluorescence imaging using appropriate filters (NF-κB, DAPI). F4/80 and 8-oxoG stained slides were counterstained with Vector hematoxylin (Vector Biolabs, Burlingame, CA) and NF-κB stained slides were counterstained with DAPI to visualize nuclei. For 8-oxoG staining, eWAT sections from *Ogg1*^−/−^ mice were used as positive controls. Negative controls were generated by incubating slides without any primary antibody. Western blots were performed at least twice with proteins from 6 animals per cohort, and IHCs were performed on two sections from each of 6 animals per cohort.

### Lipid analyses

Hepatic lipids were extracted by a modified Folch method and separated by thin-layer chromatography, as previously described^29,67^. Briefly, 20 mg of liver tissue was homogenized in chloroform:methanol (2:1), followed by phase separation by addition of acidified saline and centrifugation. The lipid fraction was dried, reconstituted in chloroform:methanol (2:1), spotted onto Silica Gel 60 chromatography plates, and developed in heptane:isopropyl ether:acetic acid (60:40:3) until the solvent front reached 1 cm from the top of the plate. Plates were dried, dipped in 10% copper sulfate in 10% phosphoric acid, air dried and charred at 110 °C for 30 minutes. Densitometry of bands corresponding to cholesterol esters, triglycerides, and diacylglycerols was performed in ImageJ.

### Transmission electron microscopy (TEM)

TEM analyses were carried out in collaboration with the Rutgers Microscopy Core. Briefly, fresh tissue sections were fixed by mincing in 2.5% glutaraldehyde / 4% paraformaldehdyde in 0.1 M cacodylate buffer and then post-fixed in buffered 1% osmium tetroxide. Samples were subsequently dehydrated in a graded series of acetone and embedded in Embed812 resin. 90 nm thin sections were cut on a Leica UC6 ultramicrotome and stained with saturated solution of uranyl acetate and lead citrate. Images were captured with an AMT (Advanced Microscopy Techniques) XR111 digital camera at 80Kv on a Philips CM12 transmission electron microscope.

### Measurement of mitochondrial respiration in eWAT

Respiration (oxygen consumption rates, OCR) in mouse eWAT was measured using XF24 extracellular flux analyzer (Seahorse Bioscience) using a protocol described by Dunham-Snary *et al*.^68^. Briefly, mouse eWAT from 7–11 weeks old animals (age matched WT and *Ogg1*^*Tg*^, n = 4) was collected and washed in rinse media (DMEM with 25 mM glucose and 25 mM HEPES, pH 7.4). Fat pads from each animal were sampled 10 times using a 2 mm UniCore Harris Punch. The tissue pieces were washed in rinse media and applied into XF24 Islet Capture Microplate (Seahorse Bioscience) resulting in ten replicates per animal and 40 replicates per genotype. Tissue pieces in the plate were rinsed two times with rinse media and two times with running media (XF DMEM base with 25 mM glucose, 1 mM pyruvate, 2 mM glutamine, pH 7.4). The final volume of running media was 500 μl per well. The plate was immediately loaded into the XF analyzer, and measurements were started. The protocol consisted of 10 cycles of basal oxygen consumption measurements followed by injection of the ATP synthase inhibitor oligomycin (10 μM), inner membrane uncoupler carbonyl cyanide 4-(trifluoromethoxy)phenylhydrazone (FCCP, 10 μM) and inhibitors of complex I and III rotenone (5 μM) and antimycin A (10 μM) to assesses various parameters of mitochondrial respiration^69^. The concentrations of inhibitors were determined via previous titration experiments. Data are expressed as pmol O_2_/min. The following parameters were calculated: basal respiration (baseline respiration minus non-mitochondrial respiration), ATP turnover-linked respiration (oligomycin-sensitive respiration), maximal respiration (maximal uncoupled respiration minus non-mitochondrial respiration), spare respiratory capacity (maximal uncoupled respiration minus basal respiration) and proton leak (oligomycin-insensitive respiration minus non-mitochondrial respiration).

### Statistical analyses

Data are expressed as mean ± SEM for biological replicates with comparisons carried out using student’s t-test for two-group comparisons or one-way ANOVA followed by post-hoc analysis using a multiple comparison procedure with Bonferroni/Dunn post-hoc comparison in Graphpad Prism. p-values < 0.05 were considered significant.

## Electronic supplementary material

Supplementary data

## Data Availability

All data generated or analysed during this study are included in this published article (and its Supplementary Information files).

## References

[1] Nakabeppu Y (2014). Cellular levels of 8-oxoguanine in either DNA or the nucleotide pool play pivotal roles in carcinogenesis and survival of cancer cells. International journal of molecular sciences.

[2] Audebert M (2000). Alterations of the DNA repair gene OGG1 in human clear cell carcinomas of the kidney. Cancer research.

[3] KC Sagun, Cárcamo Juan M., Golde David W. (2006). Antioxidants prevent oxidative DNA damage and cellular transformation elicited by the over-expression of c-MYC. Mutation Research/Fundamental and Molecular Mechanisms of Mutagenesis.

[4] Abolhassani N (2017). Molecular pathophysiology of impaired glucose metabolism, mitochondrial dysfunction, and oxidative DNA damage in Alzheimer’s disease brain. Mechanisms of ageing and development.

[5] Sampath H, McCullough AK, Lloyd RS (2012). Regulation of DNA glycosylases and their role in limiting disease. Free Radic Res.

[6] Hazra TK (2007). Oxidative DNA damage repair in mammalian cells: a new perspective. DNA Repair (Amst).

[7] Kim HJ, Jee HJ, Yun J (2011). DNA damage induces down-regulation of PEPCK and G6P gene expression through degradation of PGC-1alpha. Acta Biochim Biophys Sin (Shanghai).

[8] Klungland A, Bjelland S (2007). Oxidative damage to purines in DNA: role of mammalian Ogg1. DNA Repair (Amst).

[9] Klungland A (1999). Accumulation of premutagenic DNA lesions in mice defective in removal of oxidative base damage. Proceedings of the National Academy of Sciences of the United States of America.

[10] Nishioka K (1999). Expression and differential intracellular localization of two major forms of human 8-oxoguanine DNA glycosylase encoded by alternatively spliced OGG1 mRNAs. Molecular biology of the cell.

[11] Radicella JP, Dherin C, Desmaze C, Fox MS, Boiteux S (1997). Cloning and characterization of hOGG1, a human homolog of the OGG1 gene of Saccharomyces cerevisiae. Proceedings of the National Academy of Sciences of the United States of America.

[12] Rosenquist TA, Zharkov DO, Grollman AP (1997). Cloning and characterization of a mammalian 8-oxoguanine DNA glycosylase. Proceedings of the National Academy of Sciences of the United States of America.

[13] Cardozo-Pelaez F, Cox DP, Bolin C (2005). Lack of the DNA repair enzyme OGG1 sensitizes dopamine neurons to manganese toxicity during development. Gene Expr.

[14] Chevillard S (1998). Mutations in OGG1, a gene involved in the repair of oxidative DNA damage, are found in human lung and kidney tumours [In Process Citation]. Oncogene.

[15] Dezor M (2011). Expression of 8-oxoguanine DNA glycosylase 1 (OGG1) and the level of p53 and TNF-alphalpha proteins in peripheral lymphocytes of patients with Alzheimer’s disease. Folia Neuropathol.

[16] Dorszewska J (2009). Expression and polymorphisms of gene 8-oxoguanine glycosylase 1 and the level of oxidative DNA damage in peripheral blood lymphocytes of patients with Alzheimer’s disease. DNA Cell Biol.

[17] Fukae J (2005). Expression of 8-oxoguanine DNA glycosylase (OGG1) in Parkinson’s disease and related neurodegenerative disorders. Acta Neuropathol.

[18] Iida T, Furuta A, Nishioka K, Nakabeppu Y, Iwaki T (2002). Expression of 8-oxoguanine DNA glycosylase is reduced and associated with neurofibrillary tangles in Alzheimer’s disease brain. Acta Neuropathol.

[19] Lu R, Nash HM, Verdine GL (1997). A mammalian DNA repair enzyme that excises oxidatively damaged guanines maps to a locus frequently lost in lung cancer. Curr Biol.

[20] Mao G (2007). Identification and characterization of OGG1 mutations in patients with Alzheimer’s disease. Nucleic Acids Res.

[21] Michaels ML, Miller JH (1992). The GO system protects organisms from the mutagenic effect of the spontaneous lesion 8-hydroxyguanine (7,8-dihydro-8-oxoguanine). J Bacteriol.

[22] Nakabeppu Y, Tsuchimoto D, Yamaguchi H, Sakumi K (2007). Oxidative damage in nucleic acids and Parkinson’s disease. J Neurosci Res.

[23] Okasaka T (2009). hOGG1 Ser326Cys polymorphism and risk of lung cancer by histological type. J Hum Genet.

[24] Paz-Elizur T (2008). DNA repair of oxidative DNA damage in human carcinogenesis: potential application for cancer risk assessment and prevention. Cancer Lett.

[25] Sakumi K (2003). Ogg1 knockout-associated lung tumorigenesis and its suppression by Mth1 gene disruption. Cancer Res.

[26] Shao C (2008). Altered 8-oxoguanine glycosylase in mild cognitive impairment and late-stage Alzheimer’s disease brain. Free radical biology & medicine.

[27] Thomas D, Scot AD, Barbey R, Padula M, Boiteux S (1997). Inactivation of OGG1 increases the incidence of G. C– > T. A transversions in Saccharomyces cerevisiae: evidence for endogenous oxidative damage to DNA in eukaryotic cells. Mol Gen Genet.

[28] Sampath H (2012). 8-Oxoguanine DNA Glycosylase (OGG1) Deficiency Increases Susceptibility to Obesity and Metabolic Dysfunction. PLoS One.

[29] Vartanian Vladimir, Tumova Jana, Dobrzyn Pawel, Dobrzyn Agnieszka, Nakabeppu Yusaku, Lloyd R. Stephen, Sampath Harini (2017). 8-oxoguanine DNA glycosylase (OGG1) deficiency elicits coordinated changes in lipid and mitochondrial metabolism in muscle. PLOS ONE.

[30] Daimon M (2009). Association of the Ser326Cys polymorphism in the OGG1 gene with type 2 DM. Biochem Biophys Res Commun.

[31] Thameem F (2010). The Ser(326)Cys Polymorphism of 8-Oxoguanine Glycosylase 1 (OGG1) Is Associated with Type 2 Diabetes in Mexican Americans. Hum Hered.

[32] Yuzefovych LV (2013). Alteration of mitochondrial function and insulin sensitivity in primary mouse skeletal muscle cells isolated from transgenic and knockout mice: role ofogg1. Endocrinology.

[33] Wang W (2011). Mitochondrial DNA damage level determines neural stem cell differentiation fate. The Journal of neuroscience: the official journal of the Society for Neuroscience.

[34] Adolph, T. E., Grander, C., Grabherr, F. & Tilg, H. Adipokines and Non-Alcoholic Fatty Liver Disease: Multiple Interactions. *International journal of molecular sciences* **18**, 10.3390/ijms18081649 (2017). 10.3390/ijms18081649 PMC5578039 28758929

[35] Escobedo Noelia, Oliver Guillermo (2017). The Lymphatic Vasculature: Its Role in Adipose Metabolism and Obesity. Cell Metabolism.

[36] Frasca D, Blomberg BB (2017). Adipose Tissue Inflammation Induces B Cell Inflammation and Decreases B Cell Function inAging. Frontiers in immunology.

[37] Reilly Shannon M., Saltiel Alan R. (2017). Adapting to obesity with adipose tissue inflammation. Nature Reviews Endocrinology.

[38] Stolarczyk E (2017). Adipose tissue inflammation in obesity: a metabolic or immune response?. Current opinion in pharmacology.

[39] Pfluger PT, Herranz D, Velasco-Miguel S, Serrano M, Tschop MH (2008). Sirt1 protects against high-fat diet-induced metabolic damage. Proceedings of the National Academy of Sciences of the United States of America.

[40] Costford SR, Chaudhry SN, Crawford SA, Salkhordeh M, Harper ME (2008). Long-term high-fat feeding induces greater fat storage in mice lacking UCP3. American journal of physiology. Endocrinology and metabolism.

[41] Costford SR, Chaudhry SN, Salkhordeh M, Harper ME (2006). Effects of the presence, absence, and overexpression of uncoupling protein-3 on adiposity and fuel metabolism in congenic mice. American journal of physiology. Endocrinology and metabolism.

[42] Senese R (2011). Uncoupling protein 3 expression levels influence insulin sensitivity, fatty acid oxidation, and related signaling pathways. Pflugers Archiv: European journal of physiology.

[43] Busiello RA, Savarese S, Lombardi A (2015). Mitochondrial uncoupling proteins and energy metabolism. Frontiers in physiology.

[44] Liesa M, Shirihai OS (2013). Mitochondrial dynamics in the regulation of nutrient utilization and energy expenditure. Cell metabolism.

[45] Torres-Gonzalez M, Gawlowski T, Kocalis H, Scott BT, Dillmann WH (2014). Mitochondrial 8-oxoguanine glycosylase decreases mitochondrial fragmentation and improves mitochondrial function in H9C2 cells under oxidative stress conditions. American journal of physiology. Cell physiology.

[46] Furihata C (2015). An active alternative splicing isoform of human mitochondrial 8-oxoguanine DNA glycosylase (OGG1). Genes and environment: the official journal of the Japanese Environmental Mutagen Society.

[47] Lia Debora, Reyes Aurelio, de Melo Campos Julliane Tamara Araújo, Piolot Tristan, Baijer Jan, Radicella J. Pablo, Campalans Anna (2018). Mitochondrial maintenance under oxidative stress depends on mitochondrially localised α-OGG1. Journal of Cell Science.

[48] Takao M, Aburatani H, Kobayashi K, Yasui A (1998). Mitochondrial targeting of human DNA glycosylases for repair of oxidative DNA damage. Journal of cell science.

[49] Zhang H (2011). Obesity and hepatosteatosis in mice with enhanced oxidative DNA damage processing in mitochondria. The American journal of pathology.

[50] Panduri V (2009). Role of mitochondrial hOGG1 and aconitase in oxidant-induced lung epithelial cell apoptosis. Free radical biology & medicine.

[51] Rachek LI (2002). Conditional targeting of the DNA repair enzyme hOGG1 into mitochondria. The Journal of biological chemistry.

[52] Rachek LI, Musiyenko SI, LeDoux SP, Wilson GL (2007). Palmitate induced mitochondrial deoxyribonucleic acid damage and apoptosis in l6 rat skeletal muscle cells. Endocrinology.

[53] Rachek LI, Thornley NP, Grishko VI, LeDoux SP, Wilson GL (2006). Protection of INS-1 cells from free fatty acid-induced apoptosis by targeting hOGG1 to mitochondria. Diabetes.

[54] Yuzefovych L, Wilson G, Rachek L (2010). Different effects of oleate vs. palmitate on mitochondrial function, apoptosis, and insulin signaling in L6 skeletal muscle cells: role of oxidative stress. Am J Physiol Endocrinol Metab.

[55] Yuzefovych LV, Solodushko VA, Wilson GL, Rachek LI (2012). Protection from palmitate-induced mitochondrial DNA damage prevents from mitochondrial oxidative stress, mitochondrial dysfunction, apoptosis, and impaired insulin signaling in rat L6 skeletal muscle cells. Endocrinology.

[56] Bjorge MD (2015). Synergistic Actions of Ogg1 and Mutyh DNA Glycosylases Modulate Anxiety-like Behavior in Mice. Cell reports.

[57] Ba X, Boldogh L (2017). 8-Oxoguanine DNA glycosylase 1: Beyond repair of the oxidatively modified base lesions. Redox biology.

[58] Zarakowska E, Gackowski D, Foksinski M, Olinski R (2014). Are 8-oxoguanine (8-oxoGua) and 5-hydroxymethyluracil (5-hmUra) oxidatively damaged DNA bases or transcription (epigenetic) marks? *Mutation research*. Genetic toxicology and environmental mutagenesis.

[59] Pastukh V (2015). An oxidative DNA “damage” and repair mechanism localized in the VEGF promoter is important for hypoxia-induced VEGF mRNA expression. American journal of physiology. Lung cellular and molecular physiology.

[60] Maltseva DV, Baykov AA, Jeltsch A, Gromova ES (2009). Impact of 7,8-dihydro-8-oxoguanine on methylation of the CpG site by Dnmt3a. Biochemistry.

[61] Valinluck V (2004). Oxidative damage to methyl-CpG sequences inhibits the binding of the methyl-CpG binding domain (MBD) of methyl-CpG binding protein 2 (MeCP2). Nucleic acids research.

[62] Weitzman SA, Turk PW, Milkowski DH, Kozlowski K (1994). Free radical adducts induce alterations in DNA cytosine methylation. Proceedings of the National Academy of Sciences of the United States of America.

[63] Perillo B (2008). DNA oxidation as triggered by H3K9me2 demethylation drives estrogen-induced gene expression. Science (New York, N.Y.).

[64] Ba X (2014). 8-oxoguanine DNA glycosylase-1 augments proinflammatory gene expression by facilitating the recruitment of site-specific transcription factors. The Journal of biological chemistry.

[65] Jamaluddin M, Wang S, Boldogh I, Tian B, Brasier AR (2007). TNF-alpha-induced NF-kappaB/RelA Ser(276) phosphorylation and enhanceosome formation is mediated by an ROS-dependent PKAc pathway. Cellular signalling.

[66] Sampath H (2011). Variable penetrance of metabolic phenotypes and development of high-fat diet-induced adiposity in NEIL1-deficient mice. Am J Physiol Endocrinol Metab.

[67] Sampath Harini, Flowers Matthew T., Liu Xueqing, Paton Chad M., Sullivan Ruth, Chu Kiki, Zhao Minghui, Ntambi James M. (2009). Skin-specific Deletion of Stearoyl-CoA Desaturase-1 Alters Skin Lipid Composition and Protects Mice from High Fat Diet-induced Obesity. Journal of Biological Chemistry.

[68] Dunham-Snary KJ, Sandel MW, Westbrook DG, Ballinger SW (2014). A method for assessing mitochondrial bioenergetics in whole white adipose tissues. Redox biology.

[69] Brand MD, Nicholls DG (2011). Assessing mitochondrial dysfunction in cells. The Biochemical journal.

