# Supplementary material for: The DNA Repair Protein OGG1 Protects Against Obesity by Altering Mitochondrial Energetics in White Adipose Tissue
Source: Sci Rep. 2018 Oct 5;8:14886. doi: 10.1038/s41598-018-33151-1 (PMC6173743; doi:10.1038/s41598-018-33151-1)
Supplement: Supplementary file 1 — Supplementary data [file 41598_2018_33151_MOESM1_ESM.pdf]

The DNA Repair Protein OGG1 Protects Against Obesity by Altering Mitochondrial Energetics in White Adipose Tissue

Sai Santosh Babu Komakula[a,b], Jana Tumova[a], Deeptha Kumaraswamy[a], Natalie Burchat[a], Vladimir Vartanian[c], Hong Ye[a], Agnieszka Dobrzyn[b], R. Stephen Lloyd[c], Harini Sampath[a,*]

Supplementary Information

Supplementary Figure S1. Tissue panel of gene expression and PGC-1α protein. Expression of genes related to lipid and energy metabolism was determined by qRT-PCR using gene-specific primers (S1a). PGC-1α protein was determined by immunoblotting using a commercially available antibody (S1b). *, $p<0.05$ vs. WT. *Cpt-1, carnitine palmitoyl transferase-1, eWAT, epididymal white adipose tissue, Hsl, hormone-sensitive lipase, Mfn, mitofusin,Pgc-1 α, PPAR-gamma coactivator-1 alpha, Sirt1, sirtuin 1, Ucp, uncoupling protein.*

Supplementary Figure S2. 8-oxoG immunohistochemistry in eWAT. Immunohistochemical detection of 8-oxoG was performed using an anti-8oxoG antibody, followed by hematoxylin counterstaining. Negative controls were generated by incubating sections without primary antibody. Positive controls were generated by incubating eWAT sections from *Ogg1*$^{-/-}$ mice with 8-oxoG antibody. Images are representative of 6 animals per cohort. Scale bars represent 50 μM.

S1a

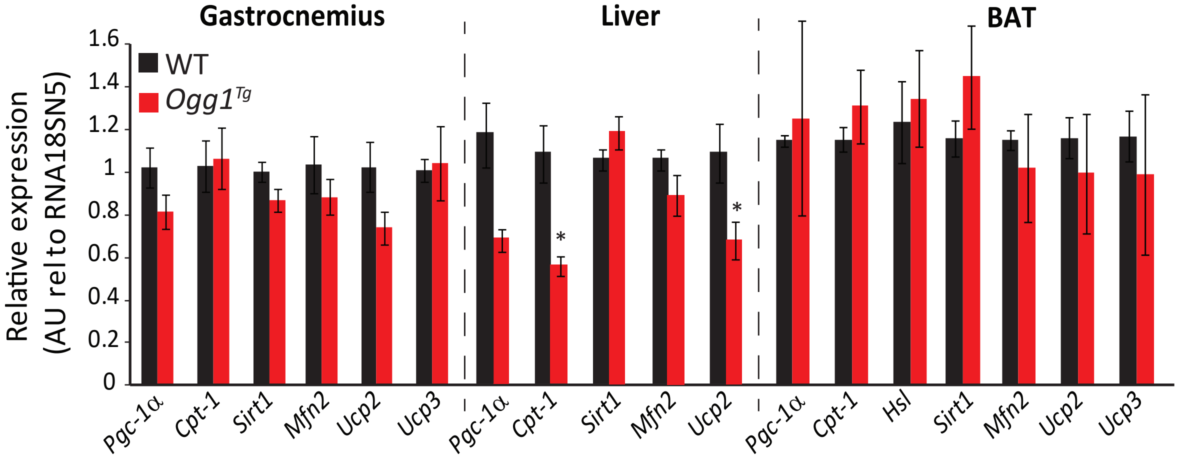
Gastrocnemius
Liver
BAT
WT
Ogg1Tg
Relative expression
(AU rel to RNA18SN5)
1.6
1.4
1.2
1
0.8
0.6
0.4
0.2
0
Pgc-1α
Cpt-1
Sirt1
Mfn2
Ucp2
Ucp3
Pgc-1α
Cpt-1
Sirt1
Mfn2
Ucp2
Pgc-1α
Cpt-1
Hsl
Sirt1
Mfn2
Ucp2
Ucp3
*
*

S1b

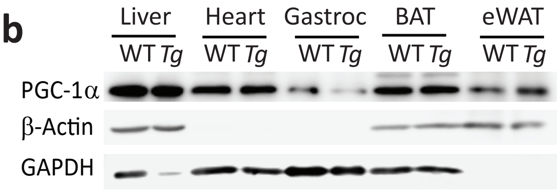
Liver
Heart
Gastroc
BAT
eWAT
WT Tg
WT Tg
WT Tg
WT Tg
WT Tg
PGC-1α
β-Actin
GAPDH

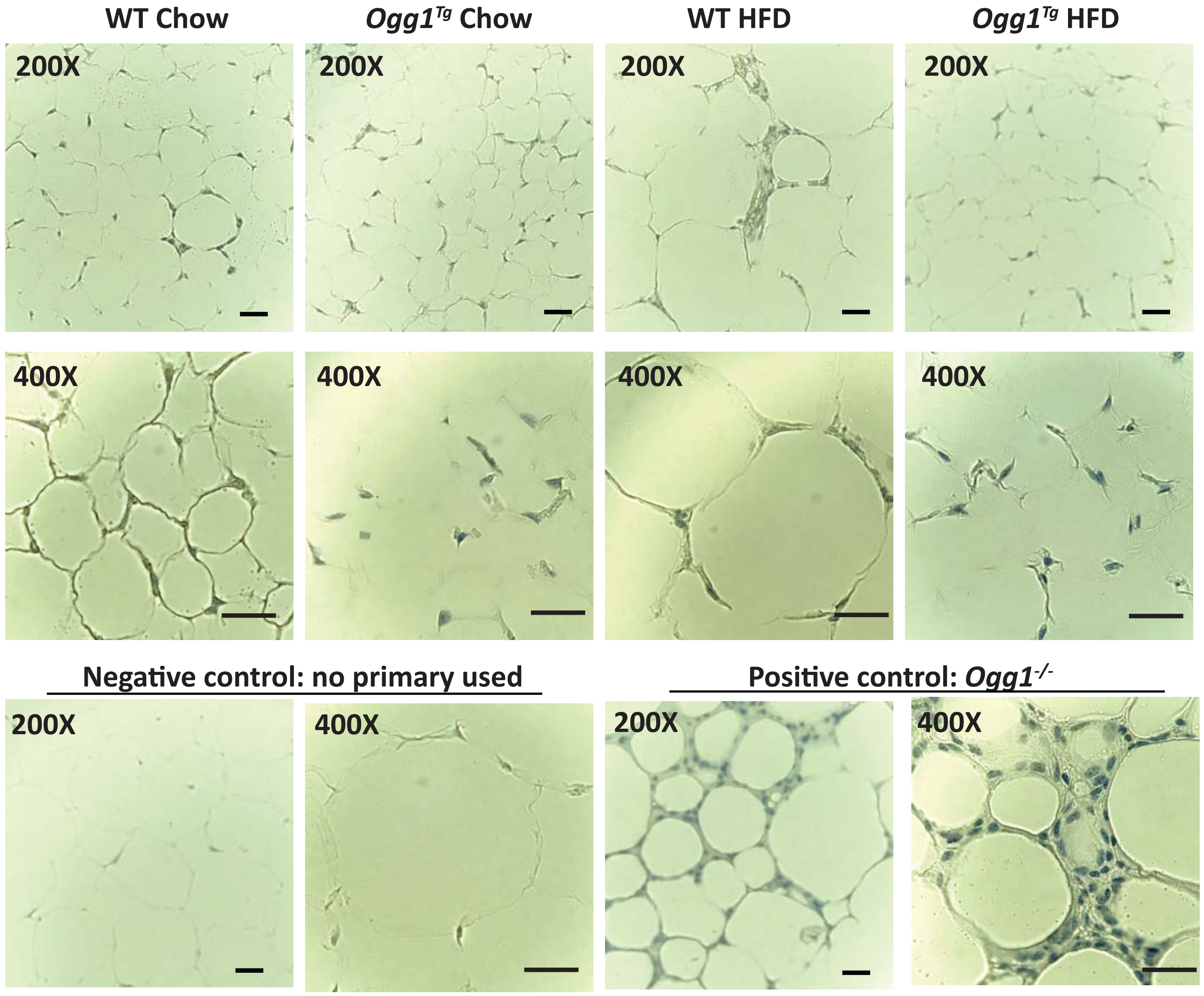
WT Chow
Ogg1Tg Chow
WT HFD
Ogg1Tg HFD
200X
200X
200X
200X
400X
400X
400X
400X
Negative control: no primary used
Positive control: Ogg1-/-
200X
400X
200X
400X
